# Successful Rehabilitation of Partial Edentulous Maxilla and Mandible with New Type of Implants: Molecular Precision Implants

**DOI:** 10.1155/2014/307364

**Published:** 2014-11-19

**Authors:** Matteo Danza, Dorina Lauritano, Francesco Carinci

**Affiliations:** ^1^University of Chieti-Pescara, Via Carducci 83, 65122 Pescara, Italy; ^2^Translational Medicine and Surgery Department, Neuroscience Centre of Milan (NeuroMI), University of Milano-Bicocca, Via Cadore 48, 20052 Monza, Italy; ^3^Chair of Maxillofacial Surgery, Department of Morphology, Surgery and Experimental Medicine, University of Ferrara, Via Luigi Borsari 46, 44121 Ferrara, Italy

## Abstract

The extraction of teeth results in rapid bone resorption both vertically and horizontally in the first month. The loss of alveolar ridge reduces the chance of implant rehabilitation. Atraumatic extraction, implant placement in extraction socket, and an immediate prosthesis have been proposed as alternative therapies to maintain the volume and contours tissue and reduce time and cost of treatment. The immediate load of implants is a universally practiced procedure; nevertheless a successful procedure requires expertise in both the clinical and the reconstructive stages using a solid implant system. Excellent primary stability and high bone-implant contact are only minimal requirements for any type of implant procedure. In this paper we present a case report using a new type of implants. The new type of implants, due to its sophisticated control system of production, provides to the implantologist a safe and reliable implant, with a macromorphology designed to ensure a close contact with the surrounding bone.

## 1. Introduction

The popularity of postextraction immediate loading implants has increased considerably between patients and dentists in the last years. The advantages of immediate loading postextraction implants surgery are evident. In fact extraction of teeth results in rapid bone resorption both vertically and horizontally in the first month [1]. The loss of alveolar ridge reduces the chance of implant rehabilitation, so implants insertion in postextractive surgery represents a solution to the loss of bone [2–4]. Bone loss is an important problem limiting implants placement for conspicuous resorption after extraction of teeth. The thinning of the ridges, the changes in gingival contour, and the loss of interdental papilla with the appearance of unsightly black spaces are the characteristics observed in these cases. The atraumatic extraction, implant placement in extraction socket, and an immediate prosthesis have been proposed as alternative therapies to maintain the volume and contours tissue and reduce time and cost of treatment [5, 6]. The maintenance of the ridges bones during the extraction, the primary stability of the implant, a careful control of the soft tissues, and proper manufacturing of the provisional prosthesis are important factors for long-term clinical success [7–9]. A proper control of biofilm with good oral hygiene during the healing period is considered a key factor in the positive outcome for the postextraction implants [10]. Over the past decade changes in dental implant design and surface configuration and an improved understanding of the biological and biomechanical aspects have improved clinical outcome of implant treatments. The ultimate goal of an improved protocol is to reduce the number of surgeries and decrease timeframe between surgery and prosthesis. These new protocols will result in increased acceptance of implant therapy. Because implant macrogeometry/microgeometry play a crucial role during the healing phase, it is important when documenting immediate loading cases to identify clearly type of implant and rehabilitation used [11, 12].

## 2. Molecular Precision Implant (MPI) Characteristics

MPI (molecular precision implants, Ditron Dental, Israel), due to its sophisticated control system of the surfaces, provides to the implantologist a safe and reliable implant, with a macromorphology designed to ensure a close contact with the surrounding bone.

The characteristics of these new implants are as follows:

MolecuLock TM:

- seal between implant and abutment,
- biomechanical design and 1 micron level production to reduce microgaps and micromovement risks;

surface treatment:

- Al_2_O_3_ surface blasting and double acid etching,
- high purity cleaning procedures;

implant body:

- high initial stability even in compromised bone situations,
- expanding tapered implant body, with double-thread self-tapping design, condensing bone gradually, to enhance primary stability,
- insertion rate of the molecular precision implants of 2.2 mm per revolution;

restorative platform:

- a beveled collar shifting the implant-abutment junction inward, in order to achieve platform-switching configuration,
- platform switching generating a perfect environment for the soft tissue growth and helping prevent bone resorption;

assisted osteointegration:

- unique spherical helix chamber forming a localized infrastructure that serves as a scaffold for promoting wound healing and bone formation from existing osteoblasts;

apex design:

- apex with self-tapping drilling blades that enables smaller osteotomy,
- the self-tapping function supporting a precise adaptation of the implant thread to the bone, thus providing optimal primary stability,
- improved ease of insertion and allowing mild direction refinement during the initial stages of insertion.

## 3. Case Report

**Figure 1:**
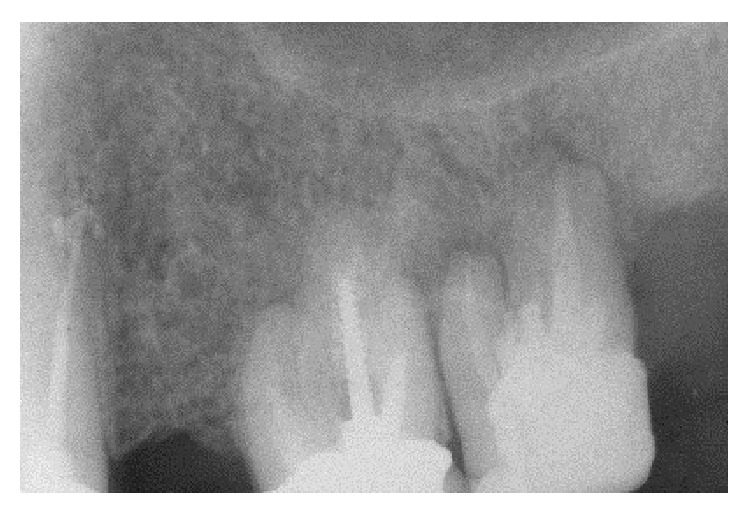
Extensive exposure of molar furcation.

A young patient aged 43 presented with pain symptoms arising during mastication at the level of the left maxillary and mandibular semiarches. X-rays showed granulomas with vertical bone reabsorption, rhizolysis, and extensive exposure of molar furcation (Figure 1).

**Figure 2:**
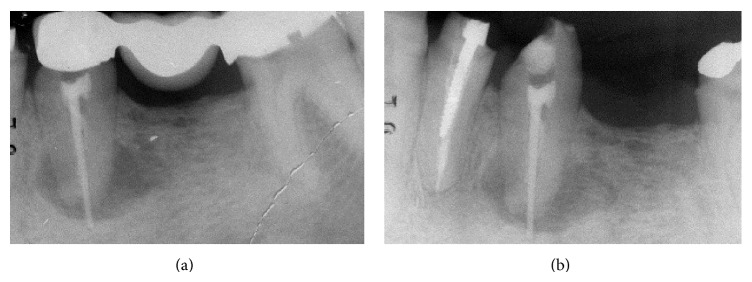
Extensive apical osseous reabsorption.

**Figure 3:**
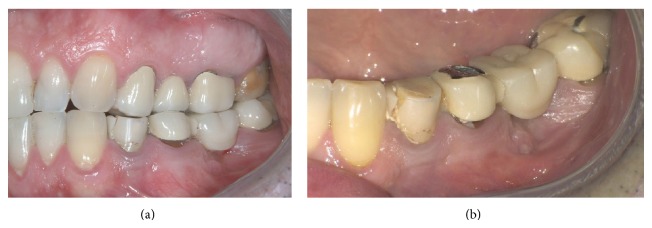
Lateral cross bite, blocking behavior, and fracture of the prosthetic crown of 3.4 and signs of suffering with gum recession in the distal teeth.

In the mandible, an extensive apical osseous reabsorption caused by endodontic cone beyond the apex was present at level 3.5 (Figures 2(a) and 2(b)). Fracture of the prosthetic crown of 3.4 and gum recession in the distal teeth (Figures 3(a) and 3(b)) were present also. The treatment plan will provide removal of the upper as well as the lower bridge, extraction of the two upper and lower molars, and postextractive immediate loading implant insertion.

**Figure 4:**
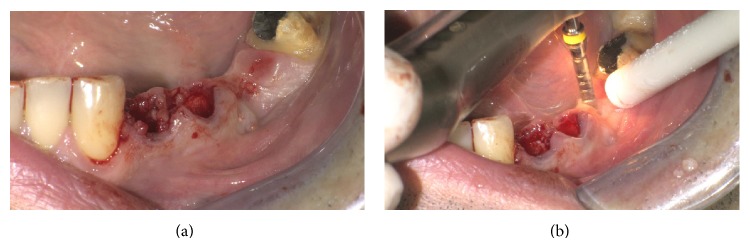
Postextractive implant site.

Atraumatic extraction of the two mandibular premolars was performed and a new site for implant in 3.6 site was formed (Figures 4(a) and 4(b)).

**Figure 5:**
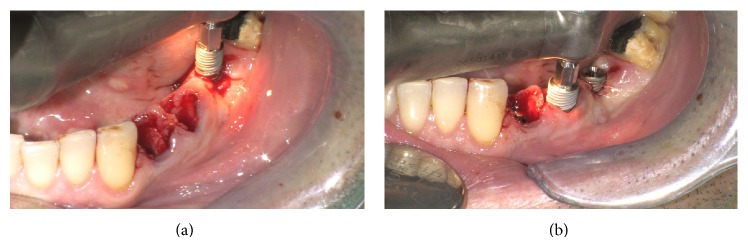
MPI 4.2 × 11.5 in 3.6 and MPI 5 × 16 in 3.5 site.

**Figure 6:**
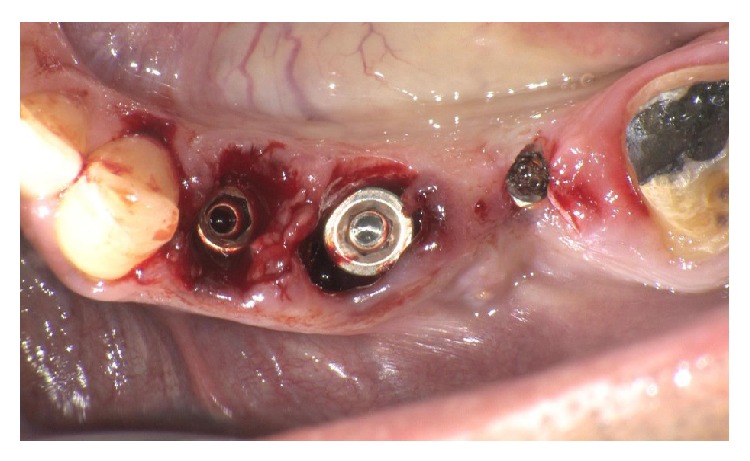
MPI 4.2 × 16 in 3.4 site.

**Figure 7:**
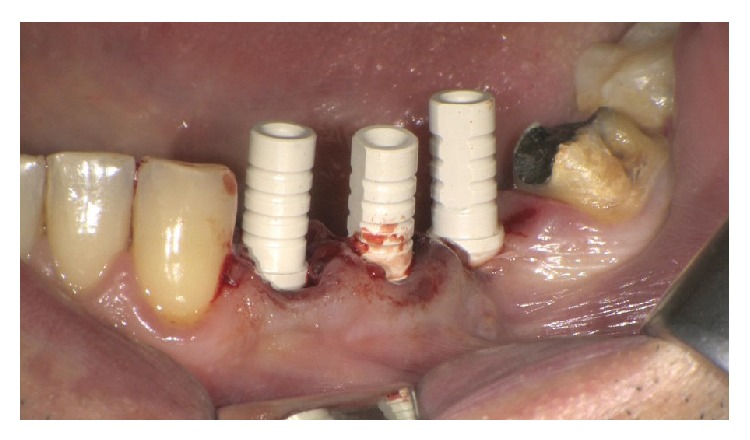
Implants placed in premolars and molar site.

**Figure 8:**
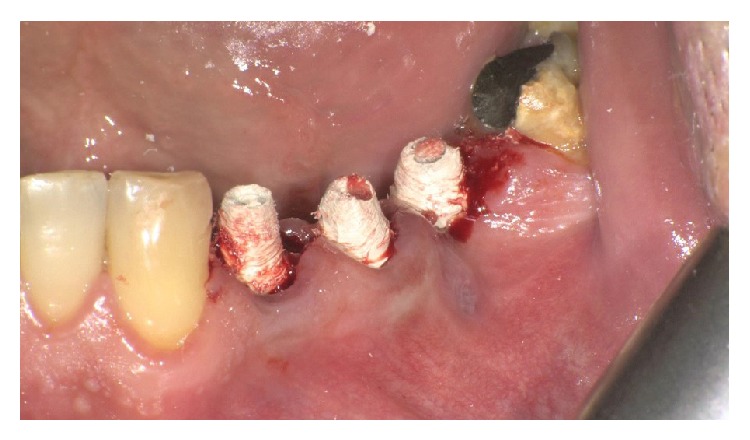
Implants with abutment.

**Figure 9:**
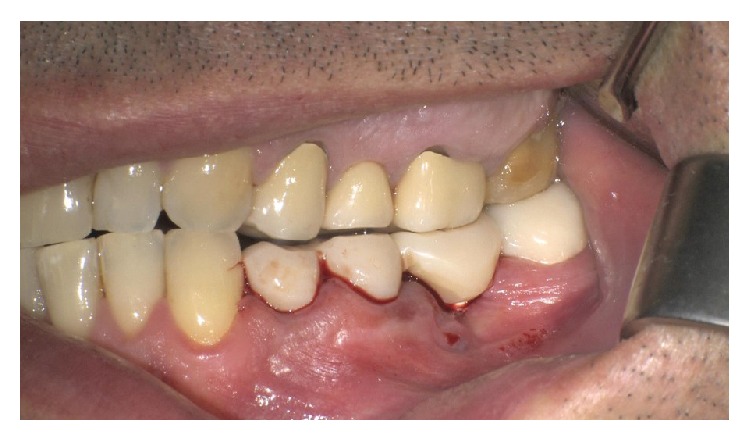
Provisional prosthesis.

**Figure 10:**
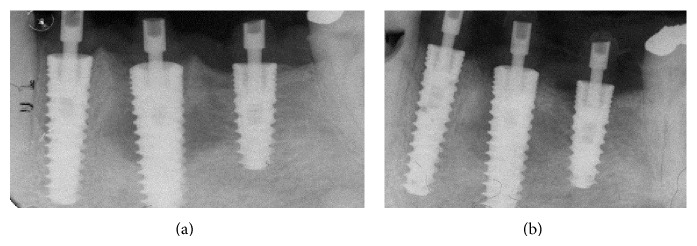
X-ray showing an optimum crestal and mesial distal position of the implants.

**Figure 11:**
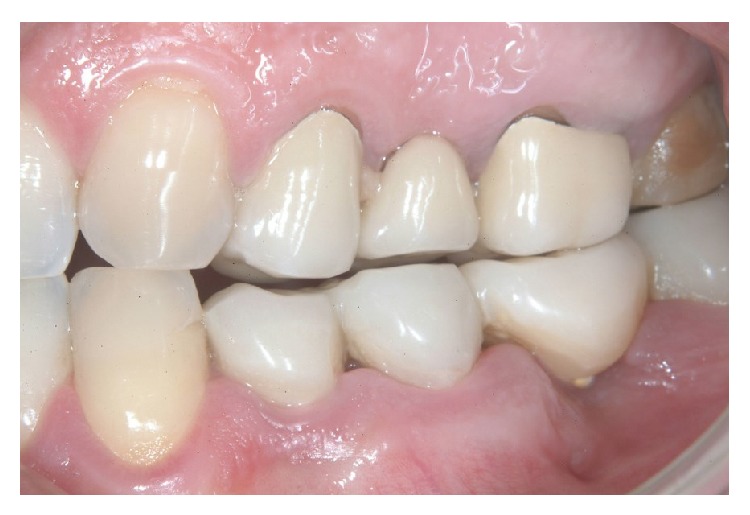
Provisional prosthesis.

MPI (Ditron Dental, Israel) were placed in different sites: 4.2 × 16 in 3.4, 5 × 16 in 3.5, and 4.2 × 11.5 in 3.6 (Figures 5(a), 5(b), and 6). All implants showed high primary stability and were loaded with provisional abutments supporting a provisional prosthetic. The X-ray showed an optimum crestal and mesial distal position of the implants, as well as their excellent position to the surrounding bone (Figures 7–11).

**Figure 12:**
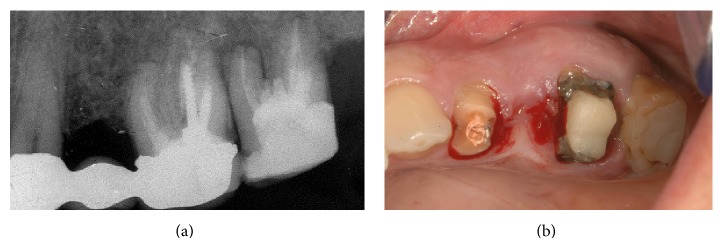
Removal of preexisting prosthesis.

**Figure 13:**
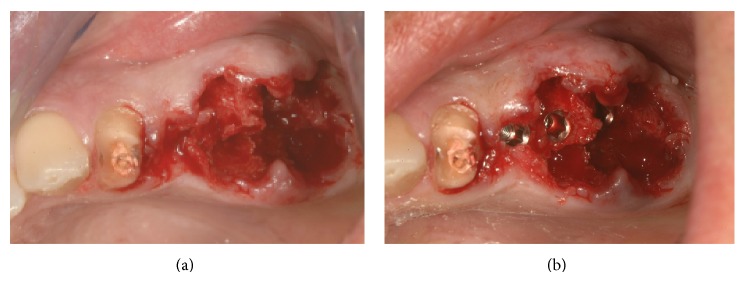
The two molars were extracted and implants positioned.

Subsequently maxilla was treated. The fixed prosthesis was removed (Figure 12(a)) and the two molars were extracted with mini-invasive technique (Figures 12(b), 13(a), and 13(b)).

**Figure 14:**
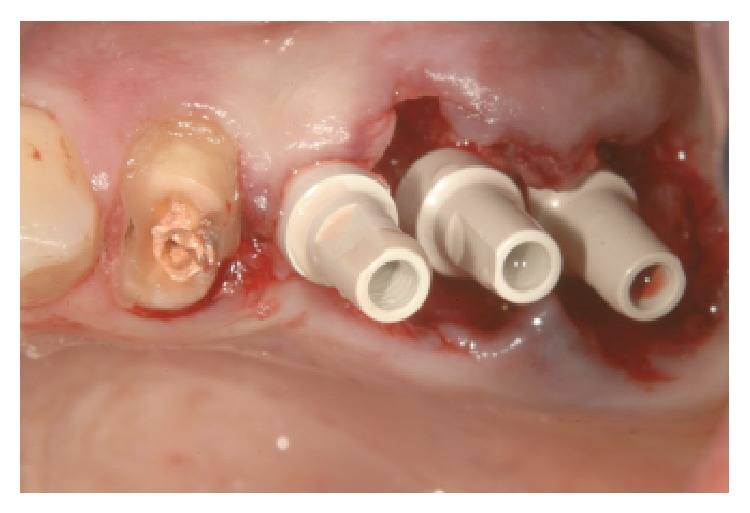
Implants inserted in 2.5, 2.6, and 2.7 sites.

**Figure 15:**
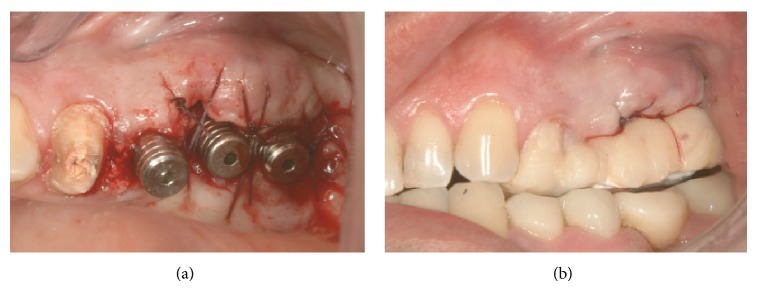
Gingival sutures and provisional prosthesis.

**Figure 16:**
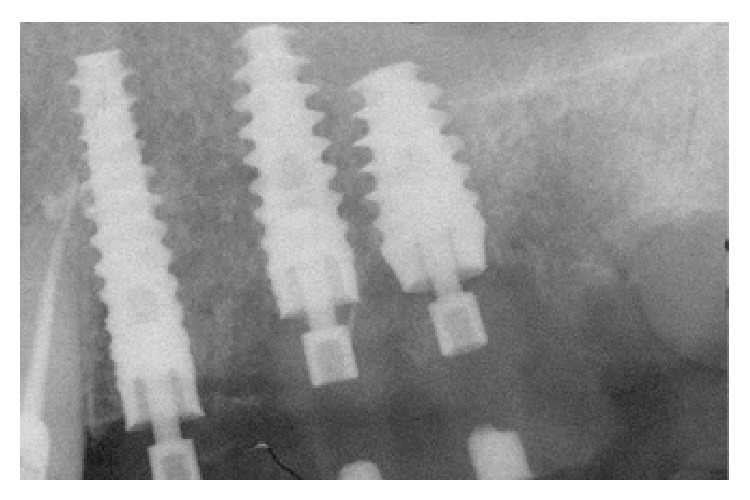
Close contact between implants and the surrounding bone.

3 MPI were inserted in correspondence to 2.5, 2.6, and 2.7 sites. The MPI measure was, respectively, 3.75 × 15, 4.2 × 13, and 6 × 10. The primary stability was excellent and the implants were immediately loaded using provisional Pek abutments (Figures 14, 15(a), 15(b), and 16).

**Figure 17:**
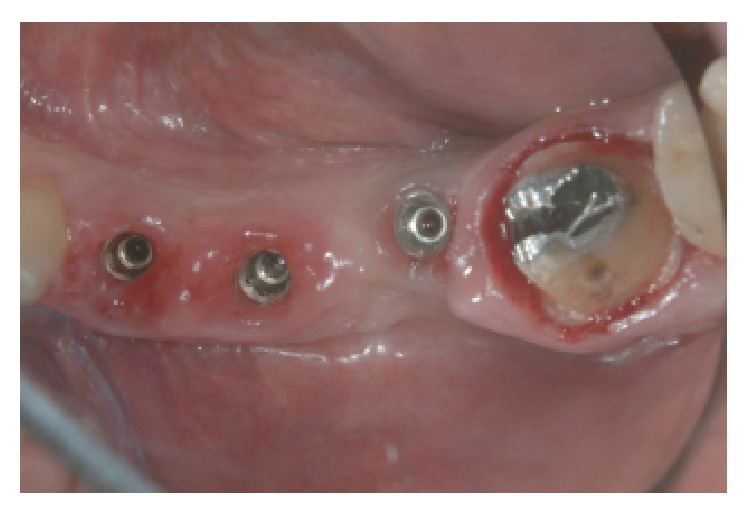
Definitive titanium abutments.

Therefore, after two months from the insertion of the maxillary implants, definitive titanium abutments were placed (Figure 17).

**Figure 18:**
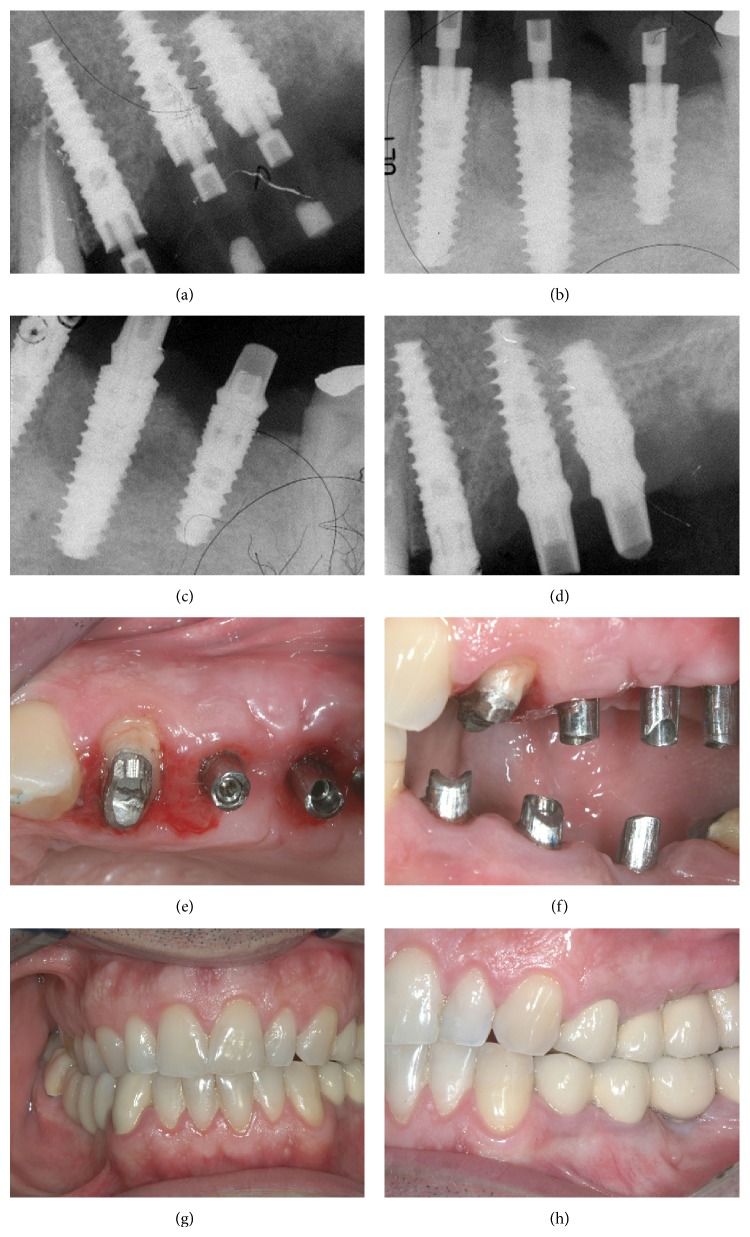
X-rays showing good implants/abutments contact and final prosthesis.

**Figure 19:**
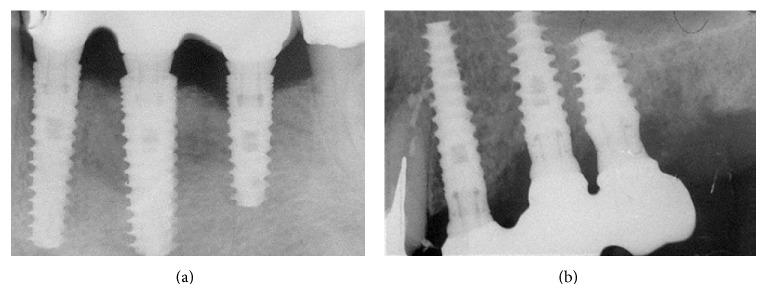
The X-ray control after final prosthesis.

X-rays showed good implants/abutments contact and final prosthesis was performed in mandibula (Figure 18) and in maxilla (Figures 19(a) and 19(b)).

## 4. Discussion

The solution of the complex implantology problems requires an accurate diagnosis. In case of postextractive immediate loading implantology the following points are requested:

- high primary stability,
- high bone-implant contact,
- reliable surface treatment.

MPI are manufactured with an extremely accurate productive process. From casting to packing, each phase is monitored to guarantee perfect manufacturing of the implant, perfect and ultra-precise prosthetic contact (MolecuLokTM), innovative surface treatment to provide a macro-micro surface geometry that improves the osteointegration process (molecular precision surface).

## 5. Conclusion

MPI (molecular precision implants) (Ditron Dental, Israel) represent the ultimate state of the art in implantology and their characteristics facilitate the placement of implants with immediate loading after extractions.
